# Combining mass spectrometry and machine learning models for predicting *Klebsiella pneumoniae* antimicrobial resistance: a multicenter experience from clinical isolates in Italy

**DOI:** 10.1186/s12866-025-04657-2

**Published:** 2026-01-19

**Authors:** Ettore Rocchi, Emanuele Nicitra, Maddalena Calvo, Valeria Cento, Laura Peiretti, Zian Asif, Giulia Menchinelli, Brunella Posteraro, Claudia Sala, Claudia Colosimo, Monica Cricca, Vittorio Sambri, Maurizio Sanguinetti, Gastone Castellani, Stefania Stefani

**Affiliations:** 1 Department of Medical and Surgical Sciences, Alma Mater Studiorum, University of Bologna, Bologna, Italy; 2 IRCCS Azienda Ospedaliero-Universitaria di Bologna, Bologna, Italy; 3 Department of Biomedical and Biotechnological Sciences, University of Catania, Catania, Italy; 4 U.O.C. Laboratory Analysis, A.O.U. Policlinico “G. Rodolico-San Marco”, Catania, Italy; 5 Department of Biomedical Sciences, Humanitas University, Via Rita Levi Montalcini 4, Pieve Emanuele, Milan, 20072 Italy; 6 IRCCS Humanitas Research Hospital, Via Manzoni 56, Rozzano, Milan, 20089 Italy; 7 Department of Laboratory and Hematology Sciences, Fondazione Policlinico Universitario A. Gemelli IRCCS, Rome, Italy; 8 Department of Basic Biotechnological, Intensive Care, and Perioperative Sciences, Università Cattolica del Sacro Cuore, Rome, Italy; 9 Precision Medicine in Clinical Microbiology Unit, Scientific Directorate, Fondazione Policlinico Universitario A. Gemelli IRCCS, Rome, Italy; 10 Unit of Microbiology, The Greater Romagna Area Hub Laboratory, Cesena, 47522 Italy

**Keywords:** Klebsiella pneumoniae, Antimicrobial resistance, Amikacin, Meropenem, Mass spectrometry, MALDI-TOF, Machine learning, Batch-effect correction, Harmonization

## Abstract

**Background:**

Multidrug-resistant *Klebsiella pneumoniae* represents a significant challenge in healthcare settings, prompting numerous studies on the rapid detection of antimicrobial resistance. Mass spectrometry has been recently integrated into routine laboratory diagnostics, providing highly sensitive results for pathogen identification. Furthermore, previously published studies have demonstrated its potential application in predicting antimicrobial resistance.

**Materials and methods:**

The study collected 686 clinical isolates of *K. pneumoniae* from three Italian hospitals and used their MALDI-TOF mass spectra as input to machine learning models for predicting susceptibility profiles to amikacin and meropenem, which were selected as the most represented antibiotic molecules within the aminoglycoside and carbapenem classes, commonly used for the treatment of *K. pneumoniae* infections. After preprocessing, *K. pneumoniae* spectra were fed to machine learning classifiers within a nested cross-validation framework. Several performance metrics were computed to compare models and identify the most appropriate one for each antibiotic. Given the multicentric nature of the study, a batch-effect correction step was applied to reduce site-specific variability using the in-house developed Python package *combatlearn* (available on GitHub: https://github.com/EttoreRocchi/combatlearn).

**Results:**

The XGBoost model achieved the best performance for both antibiotics (AUROC = 0.822 ± 0.028 for amikacin; AUROC = 0.887 ± 0.019 for meropenem). A per-site performance analysis revealed that, while performances’ variability was inherently linked to each center’s sample size, *combatlearn*-based harmonization effectively aligned mean AUROC values across sites.

**Conclusions:**

Our study demonstrates the capability of MALDI-TOF mass spectra to predict amikacin and meropenem resistance in *K. pneumoniae* directly from clinical spectra, supporting its potential as a rapid and cost-effective approach for both antimicrobial resistance surveillance through machine learning models and clinical decision support in routine microbiology practice.

**Supplementary Information:**

The online version contains supplementary material available at 10.1186/s12866-025-04657-2.

## Background

Mass spectrometry techniques have recently been integrated into clinical diagnostic workflows, representing one of the most advanced frontiers in microbiological identification. Matrix-assisted laser desorption/ionization time-of-flight mass spectrometry (MALDI-TOF MS) provides broad-spectrum pathogen identification, allowing genus- or species-level recognition within a short time frame. This analytical technique enables microbial identification from bacterial colonies or biological samples such as blood cultures, which are directly inoculated onto a platinum target plate for spectrum acquisition [1].

Despite these advances, Gram-negative pathogens remain a major healthcare challenge due to high antimicrobial resistance (AMR) rates [2]. European epidemiological surveys have reported increasing trends in β-lactam resistance among Gram-negative bacteria, particularly in *Klebsiella pneumoniae* [3]. *K. pneumoniae* is a commensal and opportunistic pathogen of the human upper respiratory tract, skin, and gastrointestinal tract. Although usually part of the normal microbiota, it can cause severe healthcare-associated infections, especially in hospitalized or immuno-compromised patients. The most frequent clinical manifestations include respiratory tract infections and bacteremia, often associated with high morbidity and mortality.

Over the past decade, the emergence and dissemination of multidrug-resistant (MDR) *K. pneumoniae* isolates, particularly those resistant to last-line antibiotics such as carbapenems, has become a global public health concern. According to recent data from the ISS–ARISS surveillance network in Italy [4], after a moderate increase in 2019–2020 and a decline during 2021–2022, carbapenem resistance (e.g., to imipenem and meropenem) in *K. pneumoniae* showed a slight rise in 2023, with cumulative resistance rates decreasing from 33.2% in 2015 to 26.5% in 2023.

Similarly, third-generation cephalosporin resistance (cefotaxime, ceftazidime, ceftriaxone) showed a minor increase in 2023, though the overall trend from 2015 to 2023 remained stable. In contrast, aminoglycoside resistance (gentamicin, amikacin) continued its gradual decline, from 42.4% in 2015 to 30.6% in 2023. However, following a reduction between 2020 and 2022, fluoroquinolone resistance (ciprofloxacin, levofloxacin) rose again in 2023 to 50.1%.

These epidemiological trends highlight the evolving dynamics of AMR in *K. pneumoniae* and emphasize the need for continuous surveillance, antimicrobial stewardship programs, and the development of new rapid methods for detecting resistance determinants, such as mass spectrometry combined with machine learning (ML).

Several studies have explored MALDI-TOF MS applications for AMR detection over the past decade [5]. Published data have demonstrated the potential of mass spectral peak analysis to identify β-lactamase-mediated hydrolysis of β-lactam antibiotics, where increasing peak intensities reflect hydrolysis products, and decreasing peaks indicate β-lactamase induction under antibiotic exposure [6–9]. Building on these principles, Oviano et al. proposed a similar approach for detecting carbapenem resistance in Gram-negative bacteria [10]. Other authors have applied a direct-on-target microdroplet growth assay to identify extended-spectrum β-lactamases (ESBLs), which involves incubating bacteria with and without antibiotics and β-lactamase inhibitors prior to MALDI-TOF analysis [11].

Moreover, Weis et al. [12] applied machine learning to MALDI-TOF spectra, demonstrating the potential of computational models for AMR classification. Together, these studies confirm the growing interest in detecting AMR through mass spectrometry-based approaches.

Based on this premise, the present study analyzes clinical isolates of *K. pneumoniae* via their MALDI-TOF spectra to predict susceptibility profiles to carbapenems and aminoglycosides using machine learning algorithms. This approach aims to advance mass spectrometry applications beyond species identification, extending its use to rapid, data-driven AMR prediction.

## Methods

### Collection of clinical isolates and corresponding spectra

The study involved three Italian hospital centers: the University Hospital “Policlinico G. Rodolico” (Catania), “Humanitas” Research Hospital (Rozzano), and the University Hospital “Fondazione Policlinico Universitario A. Gemelli” (Rome). Each center collected meropenem- and amikacin-susceptible and -resistant *K. pneumoniae* clinical isolates. These two antibiotics were selected due to their frequent inclusion in therapeutic regimens for both uncomplicated and severe infections.

Meropenem and amikacin resistance were defined as a minimum inhibitory concentration (MIC) greater than 8 mg/L, while susceptibility was defined as an MIC ≤ 2 mg/L for meropenem and < 8 mg/L for amikacin, according to the latest EUCAST guidelines [13].

Isolate identification was performed using the MALDI Biotyper^®^ Sirius System (Bruker, Billerica, MA, USA) with the MBT IVD Library (June 2021, Doc. No. 5023016). The methodology generated a mass spectrum, which was exported to the FlexAnalysis software platform (Bruker, Billerica, MA, USA). The software processed the spectral peaks, evaluating their consistency within the expected mass range.

Following FlexAnalysis processing, spectra were exported as individual.txt files for subsequent pre-processing and machine learning analyses.

### Spectra pre-processing

Mass spectra acquired via MALDI-TOF from clinical *K. pneumoniae* isolates were processed through a standardized pipeline, following the approach proposed by Weis et al. [14]. The objective of this pipeline was to extract a 6,000-dimensional feature vector from each spectrum, suitable for a machine-learning-based binary classification task.

The pre-processing phase began with a square-root transformation applied to each intensity value, a common technique to stabilize variance and reduce random noise. This step was followed by spectral smoothing using a Savitzky–Golay filter (polynomial order = 3; window size = 11) to preserve the true peak profile while attenuating high-frequency fluctuations. To remove background noise and baseline artifacts, the Statistics-sensitive Non-linear Iterative Peak-clipping (SNIP) algorithm [15] was applied.

After baseline correction, each spectrum was discretized through binning with a bin width of 3 Da – a favorable trade-off between preserving informative peaks and reducing the dimensionality of the feature set, as suggested by Weis et al. [14], considering only the m/z range from 2,000 to 20,000 Da, which is standard in clinical microbiology for bacterial species identification. The resulting feature vectors were normalized via Total Ion Count (TIC) normalization, adjusting each intensity value (I_ij_) according to the formula:

$${I_i}_j{'}={I_i}_j/(TIC_j)$$

where.

$$TIC_j\;=\sum_{i=1}^nI_{ij}$$

Here, $\mathrm{TIC_j}$ represents the total ion count of the j-th spectrum, $\mathrm{I_{ij}}$ the i-th intensity (of the i-th bin) in the j-th spectrum, and n is the total number of bins. This normalization ensures comparability across spectra with varying total signal intensities. 

All preprocessing steps were performed using the MaldiAMRKit Python package (v0.3.0), available on GitHub at https://github.com/EttoreRocchi/MaldiAMRKit.

The resulting normalized vector representation of each MALDI-TOF spectrum was then used as input for supervised classification. When aggregating spectra from all centers, a harmonization step was introduced to mitigate inter-site variability and reduce batch effects that could bias results. This was achieved through a customized implementation of the ComBat algorithm, specifically adapted for integration into the machine learning pipeline. This tool ensures harmonization without data leakage and is publicly available at https://github.com/EttoreRocchi/combatlearn. Specifically, harmonization is implemented as a transformation step within a scikit-learn *Pipeline*. Thus, ComBat parameters are fitted exclusively on the training portion of each outer cross-validation fold, and the harmonization model is subsequently applied to the corresponding validation split (within the inner cross-validation) or test split (within the outer cross-validation), so that data leakage is prevented both at the outer and inner cross-validation levels. Site-specific identifiers are the only covariates used by the ComBat model.

Finally, all feature vectors were standardized to zero mean and unit variance per feature within the training pipeline.

### Spectra analyses

MALDI-TOF spectra classification aimed at distinguishing between susceptible and resistant *K. pneumoniae* isolates was performed using multiple machine learning models, including logistic regression, random forest, XGBoost, and multilayer perceptron (MLP). Model training and evaluation were conducted within a nested cross-validation framework, consisting of a 10-fold outer loop for assessing generalization performance and a 5-fold inner loop for hyperparameter tuning. Each antibiotic was analyzed independently.

We performed two complementary analyses to comprehensively evaluate the predictive capability of MALDI-TOF mass spectra in detecting antimicrobial resistance using machine learning models. These analyses were designed to capture distinct yet complementary aspects of the data and their clinical relevance.

The first analysis considered the entire dataset, aggregating spectra from all participating hospitals. This approach maximized sample size and diversity, allowing us to assess how well harmonized models generalize across epidemiologically distinct sites. Stratification during cross-validation was performed jointly by batch and binary outcome, the latter representing either resistance or susceptibility to the antibiotic under investigation. To mitigate class imbalance, all models were trained using class-balanced weighting to avoid favoring the majority class. Batch effect correction was implemented using a ComBat-based method [16] to mitigate site-specific biases, ensuring that performance estimates reflected biological rather than technical variation. Feature importance was assessed using SHapley Additive exPlanations (SHAP) values to interpret model behavior.

The second analysis focused on site-specific modeling strategies. For each clinical center, models were trained exclusively on local data using the same nested cross-validation scheme. In addition to within-site evaluation, each model was externally tested on datasets from the other two centers, enabling assessment of cross-site generalization.

This cross-validation strategy allowed us to evaluate both local discriminative power and external robustness, providing insight into the model’s ability to generalize across different epidemiological and technical contexts. Such cross-site testing is particularly relevant for real-world deployment, where models may be applied outside the institution where they were developed.

Together, these two complementary strategies enabled the investigation of two key dimensions of model performance: (1) global robustness and scalability when using harmonized, multi-center data; and (2) local specificity and inter-site heterogeneity in resistance profiles and spectral characteristics. Combined, they offer a comprehensive understanding of the models’ practical applicability in clinical microbiology.

## Results

**Fig. 1:**
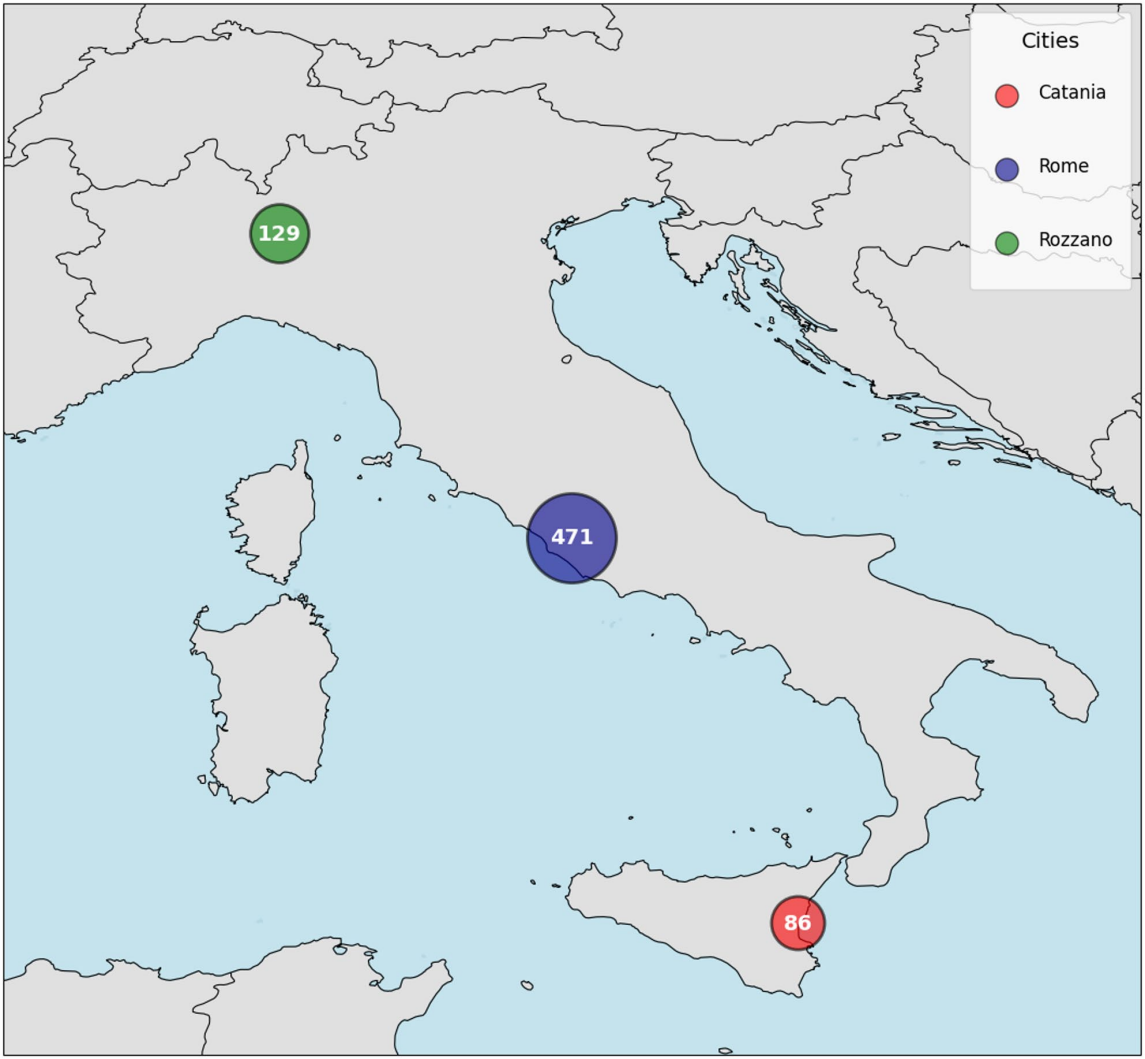
Number of *Klebsiella pneumoniae* isolates provided by the participating hospital centers

Our study included a total of 686 *K. pneumoniae* isolates, which were collected from the participating hospital centers. Catania provided 86 isolates, Rome provided 471, and Milan provided 203 isolates. Figure 1 illustrates the number of isolates provided by each center, while Table 1 summarizes the susceptibility and resistance profiles of the collected *K. pneumoniae* isolates.

**Table 1:** Summary of meropenem-susceptible, meropenem-resistant, amikacin-susceptible, and amikacin-resistant *Klebsiella pneumoniae* isolates provided by each participating hospital, along with their corresponding MIC ranges

Source	*n*° of MEM- susceptible strains	MEM-susceptibility MIC ranges (mg/L)	*n*° of MEM- resistant strains	MEM-resistance MIC ranges (mg/L)	*n*° of AK- susceptible strains	AK-susceptibility MIC ranges (mg/L)	*n*° of AK- resistant strains	AK-resistance MIC ranges (mg/L)
CATANIA	15	0.125–4.125	71	8–128	15	0.5–4.5	71	16–128
MILAN	99	0.25–4.25	30	16–64	104	4–8	25	16–32
ROME	199	0.125–128.125	272	16–128	338	2–8	133	16–128

*Abbreviations:* *MEM*, meropenem; *AK*, amikacin

The performance evaluation of models trained on MALDI-TOF spectra, corrected for batch effects using the ComBat algorithm, revealed that XGBoost consistently outperformed other classifiers across both antibiotic targets – amikacin and meropenem. The reported values represent the mean performance obtained through 10-fold external cross-validation, with corresponding 95% confidence intervals. In the nested cross-validation procedure, both inner and outer folds were stratified by site of origin and resistance class (separately for amikacin and meropenem) to ensure that each fold accurately reflected the overall distribution with respect to site and prevalence.

### The amikacin target

**Fig. 2:**
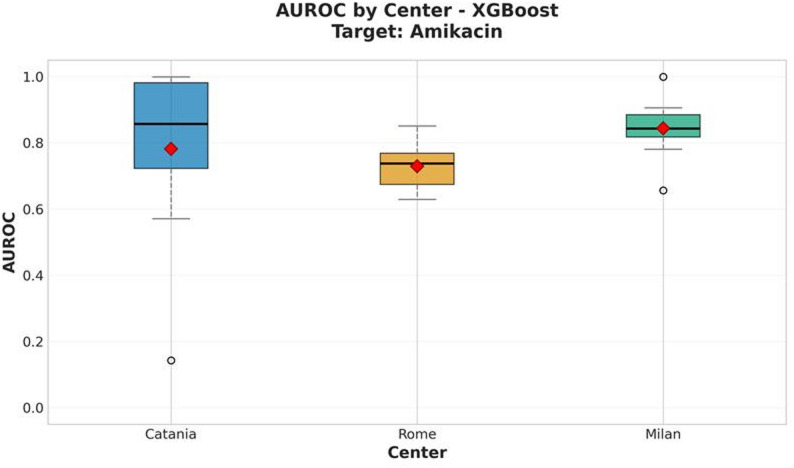
AUROC performance of the XGBoost model by center for amikacin resistance prediction. The red diamond indicates the mean value across all cross-validation iterations

**Fig. 3:**
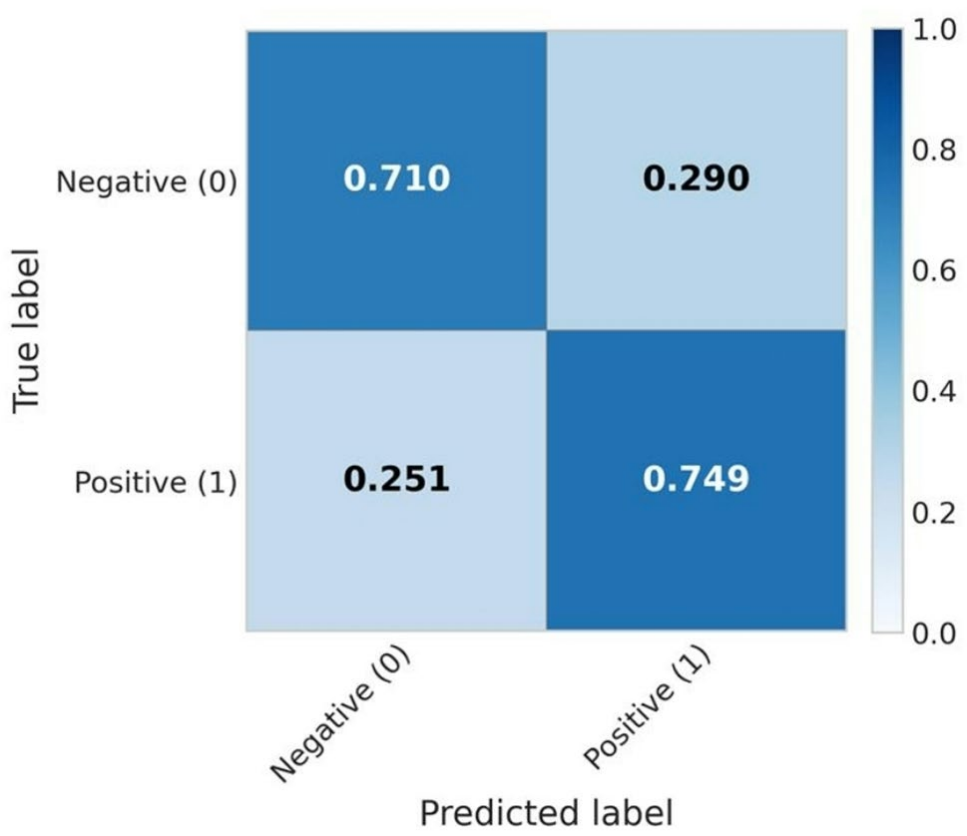
Mean normalized confusion matrix across the 10-fold cross-validation for amikacin resistance prediction

**Fig. 4:**
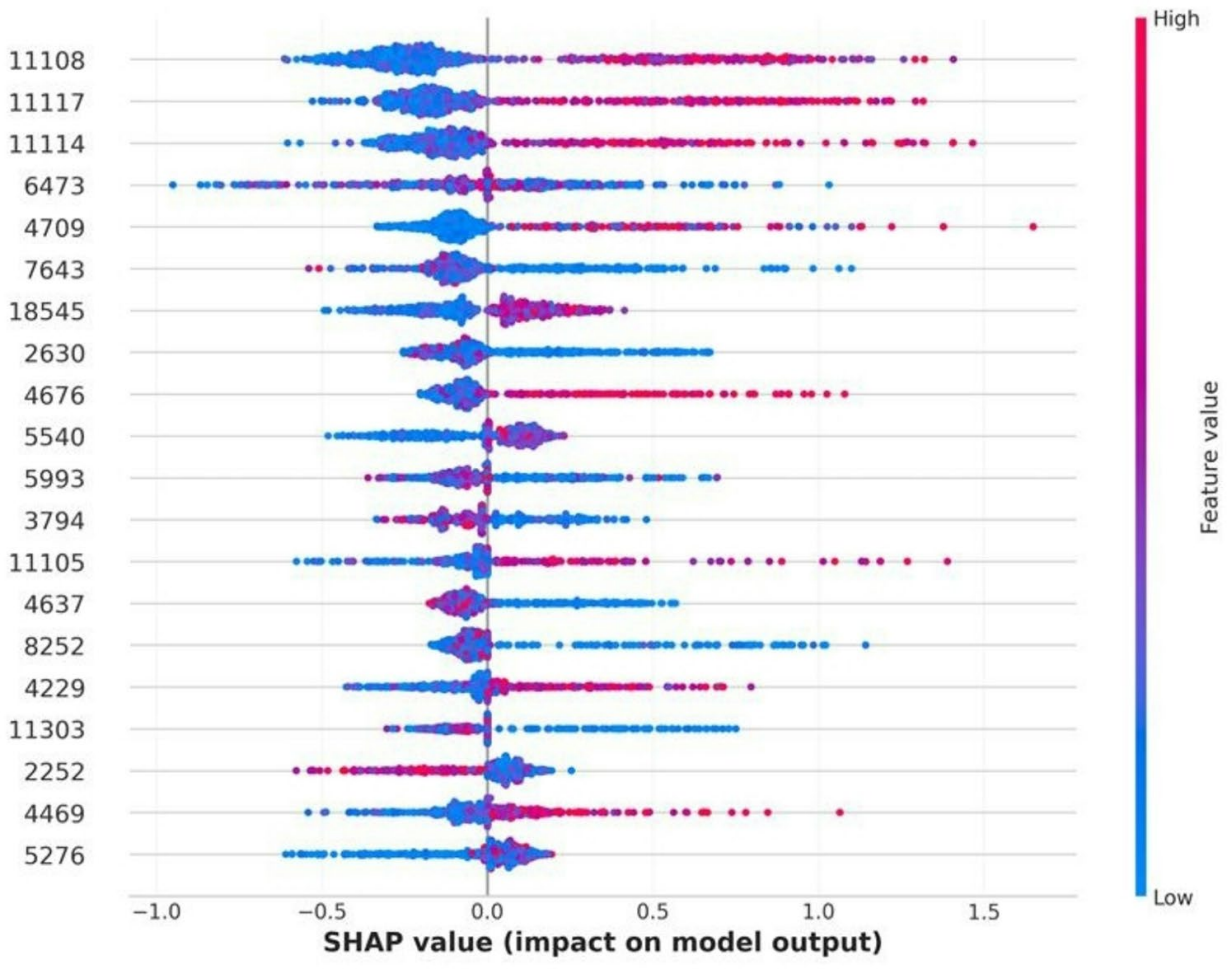
SHAP beeswarm plot showing the top contributing features influencing the XGBoost model predictions for amikacin resistance

For amikacin, the XGBoost model yielded a mean AUROC of 0.822 ± 0.028 and a balanced accuracy of 0.729 ± 0.043. Figure 2 illustrates the per-site performance distribution across the 10 test iterations of the outer cross-validation for the harmonized datasets. The confusion matrix of the XGBoost classifier is presented in Figure 3, while the SHAP beeswarm plot summarizing feature importance within the framework is shown in Figure 4.

### The meropenem target

**Fig. 5:**
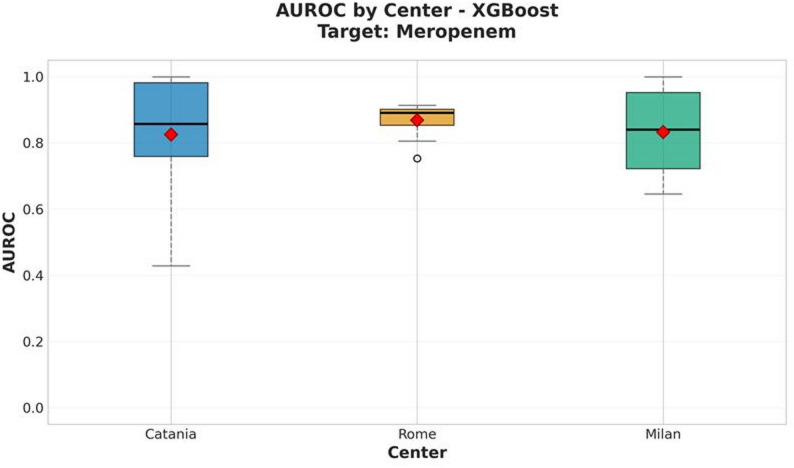
AUROC performance of the XGBoost model by center for meropenem resistance prediction. The red diamond indicates the mean value across all cross-validation iterations

**Fig. 6:**
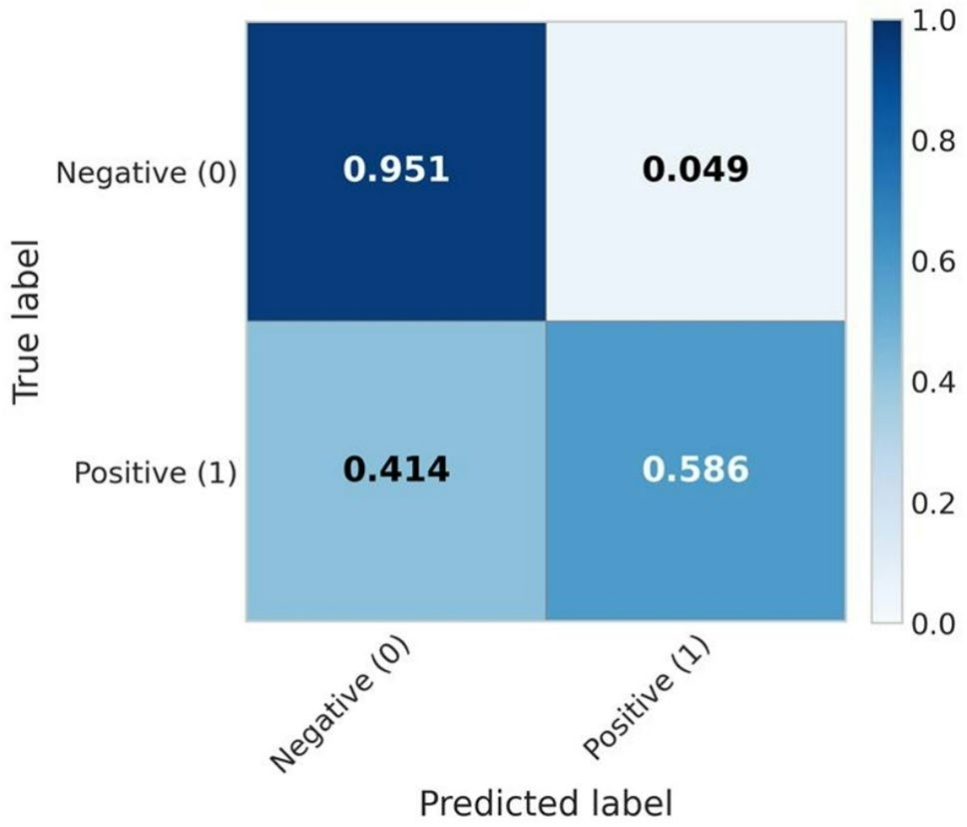
Mean normalized confusion matrix across the 10-fold cross-validation for meropenem resistance prediction

**Fig. 7:**
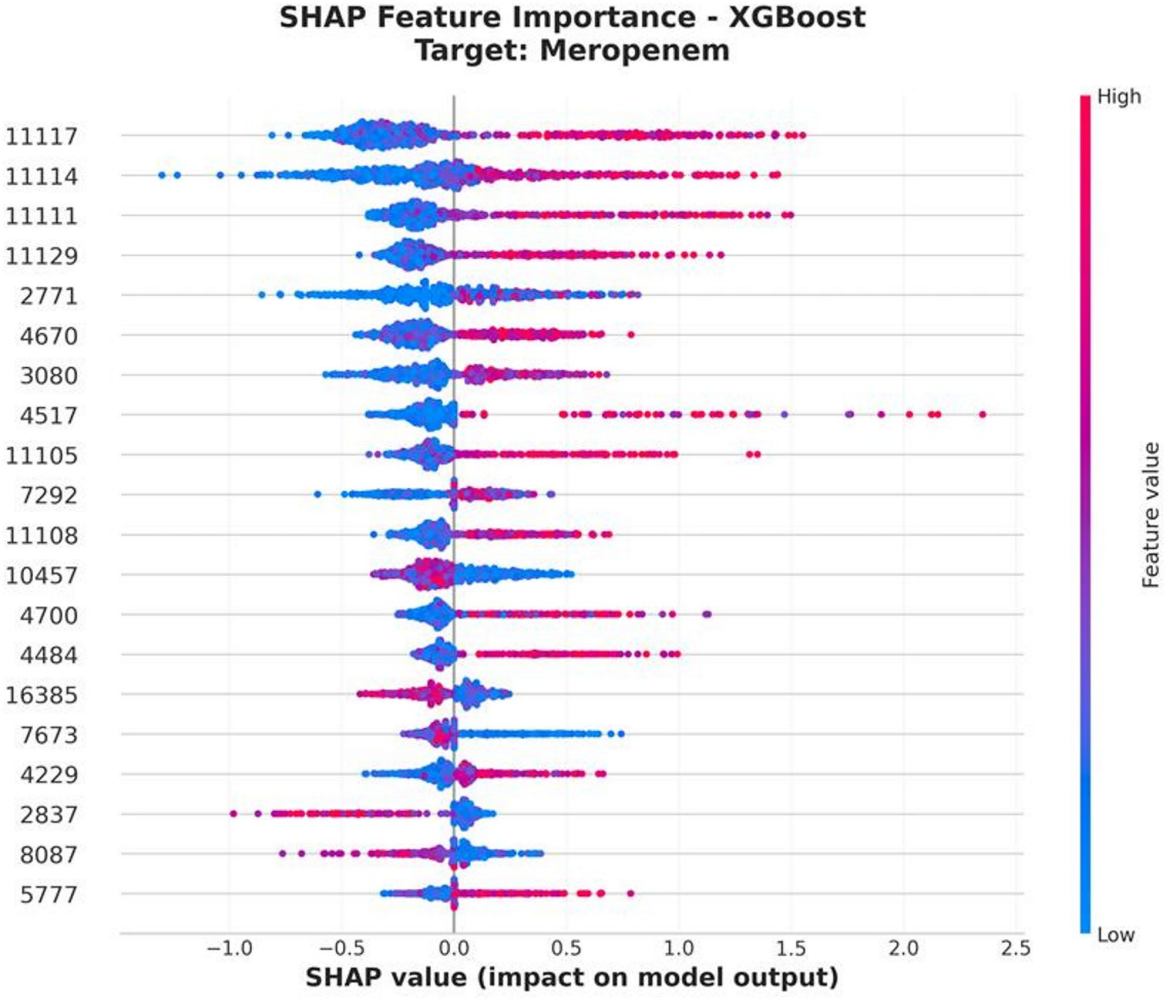
SHAP beeswarm plot showing the top contributing features influencing the XGBoost model predictions for meropenem resistance

For meropenem, the XGBoost model achieved a mean AUROC of 0.887 ± 0.019 and a balanced accuracy of 0.768 ± 0.015, indicating robust classification performance across the harmonized datasets. As for the amikacin analysis, per-site performances, the normalized confusion matrix, and the SHAP feature importance plots are presented in Figures 5, 6, and 7, respectively.

### Cross-site framework

For what concerns the cross-site evaluation, the heatmap illustrates the Area Under the Receiver Operating Characteristic (AUROC) scores obtained when training XGBoost models on data from individual clinical centers (Catania, Milan, and Rome) and evaluating them on both intra- and inter-center test sets. Each cell in the matrix represents the mean AUROC derived from a 10-fold cross-validation procedure, where models were trained exclusively on data from one center (rows) and tested on data from another – or the same – center (columns), while maintaining identical test folds across iterations to ensure a fair and consistent comparison.

Diagonal values represent within-center performance, highlighting how well each model captures local signal characteristics and resistance patterns. Notably, models trained and tested within Milan and Rome achieved strong discriminative performance (AUROC = 0.838 and 0.860, respectively), while Catania exhibited slightly lower within-site performance (AUROC = 0.792). Off-diagonal cells, on the other hand, reflect the model’s ability to generalize across sites. The generally lower AUROC values in these positions suggest a reduction in cross-center generalizability, reflecting heterogeneity in local epidemiology or instrument calibration differences. Models trained on Rome data generalized better across other centers, likely due to the larger and more diverse dataset, which provided greater variability in the training phase and thus improved robustness.

**Fig. 8:**
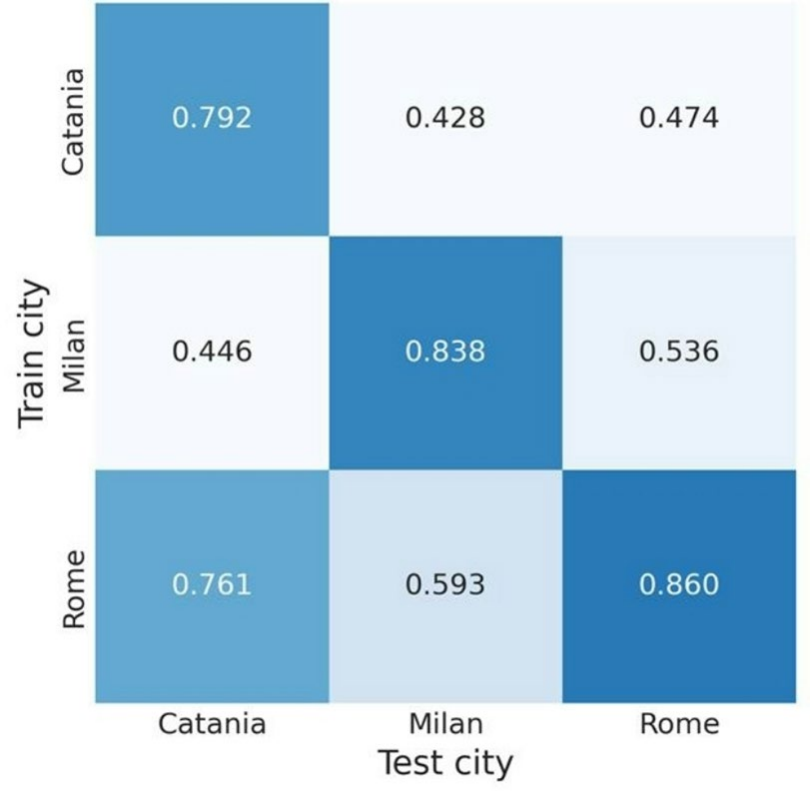
Cross-site AUROC performance of the XGBoost model for meropenem prediction

It is worth noting that the comparability of AUROC values across cells was ensured by the design of the evaluation protocol. In each iteration of the cross-validation process, test folds were kept constant, allowing a direct and fair comparison of results across centers and ensuring that observed differences reflected model generalization rather than test-set variance. A similar performance pattern was observed for amikacin, as detailed in the Supplementary Materials.These findings – summarized in Figure 8 – highlight both the opportunities and challenges of deploying predictive models across clinical centers, emphasizing the importance of harmonization or domain adaptation strategies to ensure robust performance in multi-site implementations.

### Comparative model performance summary

Regarding the comparative performance of the models, Table 2 and Table 3 summarize the balanced accuracy and AUROC values for each classifier trained on batch-corrected datasets. These values represent the mean performance across 10-fold external cross-validation, with 95% confidence intervals reported as error estimates. Detailed information on model selection, hyperparameter optimization, and additional performance metrics is provided in the Supplementary Materials.

**Table 2:** AUROC, balanced accuracy and resistance class’s recall performances of the four machine learning models trained to classify amikacin resistance and susceptibility in K. pneumoniae isolates

**Model**	**AUROC (Mean ± Std. Dev.)**	**Balanced Accuracy (Mean ± Std. Dev.)**	**Resistance class recall (Mean ± Std. Dev.)**
XGBoost	**0.822 ± 0.040**	**0.729 ± 0.060**	0.749± 0.076
Random forest	0.802 ± 0.039	0.725 ± 0.036	**0.757 ± 0.044**
MLP	0.739 ± 0.058	0.684 ± 0.061	0.692 ± 0.129
Logistic regression	0.741 ± 0.064	0.691 ± 0.067	0.642 ± 0.115

Highest results are highlighted in bold

**Table 3:** AUROC, balanced accuracy and resistance class’s recall performances of the four machine learning models trained to classify meropenem resistance and susceptibility in K. pneumoniae isolates

**Model**	**AUROC (Mean ± Std. Dev.)**	**Balanced Accuracy (Mean ± Std. Dev.)**	**Resistance class recall (Mean ± Std. Dev.)**
XGBoost	**0.887 ± 0.027**	**0.768 ± 0.022**	0.586 ± 0.044
Random forest	0.870 ± 0.036	0.673 ± 0.054	0.355 ± 0.107
MLP	0.768 ± 0.042	0.688 ± 0.050	0.465 ± 0.073
Logistic regression	0.829 ± 0.041	0.754 ± 0.044	**0.684 ± 0.061**

Highest results are highlighted in bold

## Discussion

Multidrug-resistant (MDR) *K. pneumoniae* represents a major global threat, particularly in critical care settings and high-risk epidemiological areas. Various approaches have been explored to detect its resistance mechanisms, including phenotypic, molecular, and rapid immunochromatographic assays [17]. Real-time polymerase chain reaction (PCR) technologies integrated the conventional resistance markers microbiological workflows, along with ultimate generation methods as whole genome sequencing (WGS) [18]. Moreover, microbiological laboratory routine often includes rapid phenotypic antimicrobial susceptibility testing (AST), which reveals minimum inhibitory concentration values directly from biological samples [19]. Certainly, rapid AST and molecular methods significantly reduced turn-around-time (TAT). However, they sometimes require expensive diagnostic tools and personnel expertise, limiting their potential extension to all the biological samples [17–19].

More recently, machine learning techniques have been applied to AMR prediction, showing considerable potential across both bacterial and yeast clinical isolates [14, 18, 19]. Mass spectrometry, already widely integrated into diagnostic workflows for microbial identification, further expands these possibilities. Its integration with ML could ultimately support automated, rapid AMR prediction among critical pathogens [20].

Given the widespread diffusion of MDR *K. pneumoniae* in Italy [3, 21], this multicenter study aimed to evaluate ML models for the rapid prediction of meropenem and amikacin resistance using MALDI-TOF MS spectra from clinical isolates. The three participating hospitals, located in Northern, Central, and Southern Italy, provided an ideal epidemiological representation for the study. MALDI-TOF MS spectra were analyzed using pre-processing and machine learning pipelines to identify spectral features associated with susceptibility or resistance patterns.

The superior performance of XGBoost across both antibiotics likely reflects its ability to capture complex, non-linear patterns and handle high-dimensional, collinear spectral data. MALDI-TOF spectra are inherently variable and sparse, features that XGBoost can effectively manage through boosting. The SHAP-based interpretability analysis supported these findings, highlighting specific m/z peaks that consistently contributed to resistance prediction. Furthermore, harmonization with the ComBat algorithm (implemented via the *combatlearn* framework) successfully mitigated inter-site batch effects, as confirmed by consistent AUROC values across centers (Figs. 2 and 5). This adjustment preserved biological signals while minimizing technical bias, enhancing the reproducibility and generalizability of the models in multicenter settings.

At the site level, performance stability was highest in Rome, consistent with its larger dataset, while Catania exhibited slightly lower within-site AUROC scores due to its smaller sample size, which limits the reliability of site-specific conclusions. Nonetheless, it is worth pointing out that the harmonization-based framework enabled the predictive model to achieve promising performances even at this site, regardless of the limited training contribution it provided (as expected, these performances were associated with a higher variability, linked to the small sample size and to the inherent greater heterogeneity of the available isolates). More in depth, the performance degradation in the cross-site evaluation may be attributed to different insights. First, site-specific epidemiological differences must be acknowledged, including heterogeneity in the prevalence and distribution of resistance mechanisms which can alter spectral signatures and, ultimately, hinder transferability of models trained on a single site. Second, technical heterogeneities also arise, e.g. differences in mass spectrometers’ calibration procedures. Third, the uneven sample sizes, particularly the predominance of isolates from Rome, may bias the model learning phase toward site-specific patterns, further reducing the generalization ability of models. Overall, cross-site testing confirmed that model generalization decreases when applied across centers, highlighting the importance of harmonization strategies to manage differences in local epidemiology and instrument calibration. Therefore, explicit modeling and correction of site-related batch effects within the learning pipeline enable the robustness and reliability of predictive performances and could represent a step forward clinical deployment of machine learning-based analyses of MALDI-TOF mass spectra for antimicrobial resistance prediction.

Our results align with those reported by López-Cortés et al. [22], who used CatBoost to predict ciprofloxacin-resistant *K. pneumoniae*, *Escherichia coli*, and *Staphylococcus aureus* based on MALDI-TOF spectra (AUROC = 0.73, F1 = 0.78) and similarly employed SHAP values to identify relevant m/z peaks [23]. Likewise, Nguyen et al. [24] applied AI models to *Pseudomonas aeruginosa*, using dynamic binning and transfer learning to improve cross-dataset generalizability, reporting strong performance for both β-lactam/β-lactamase inhibitors (AUROC = 0.869) and aminoglycosides such as amikacin (AUROC = 0.844) [23].

Compared with these studies, our work focuses specifically on meropenem and amikacin resistance in *K. pneumoniae*, achieving comparable or higher predictive performance. Moreover, our use of batch-effect correction through *combatlearn* demonstrates an additional methodological advancement, enabling reliable inter-site integration in ML workflows without data leakage. Regarding the achieved AUROC values (0.822 for amikacin and 0.887 for meropenem), interesting considerations may be discussed. Unfortunately, there is not a current standardization about AUROC values and their applicability into clinical and therapeutical workflows. However, clinical diagnostic investigations previously recognized the AUROC interval 0.70–0.80 as acceptable, the 0.80–90 interval as excellent, and > 0.90 values as optimal [25, 26]. According to these assumptions, we could consider as promising the values obtained within our study, hypothesizing future implementations for both the tested molecules and their antimicrobial resistance prediction rates through machine learning models. On the other hand, further studies will be essential to clarify the obtained recall values, especially regarding the meropenem analysis.

According to our data, the 0.586–0.684 meropenem recall interval may be related to possible false susceptible isolates, compromising a prompt clinical usage of the reported results.

Although the interesting data collected about meropenem and amikacin, most of the MDR *K. pneumoniae* isolates currently require the introduction of last resort antimicrobial choices. For instance, meropenem/vaborbactam demonstrated potent in vitro and in vivo activity in the case of complicated *K. pneumoniae* infections [27]. Additionally, cefiderocol and aztreonam/avibactam became fundamental therapeutical choices against Gram-negatives severe infections, especially within high-risk epidemiological areas [28]. Finally, ultimate investigations involve cefepime/taniborbactam, which showed promising efficacy against *K. pneumoniae* clinical isolates [29, 30]. These newest antimicrobial resources may be furtherly analysed through machine learning models, particularly examining the models’ evolution in presence of both the antibiotic and the β-lactamases inhibitor.

In conclusion, the integration of MALDI-TOF MS and ML represents a fast, cost-effective strategy for routine AMR surveillance in clinical microbiology. With further validation and expanded datasets, these models could be incorporated into existing laboratory workflows to provide real-time probabilistic outputs on susceptibility profiles, improving the speed and precision of antimicrobial therapy guidance.

## Supplementary Information

Supplementary Material 1.

## Data Availability

The dataset analyzed in this study is publicly available in the MALDI-Kleb-AI Zenodo repository at: 10.5281/zenodo.17405072.The data processing and analysis pipeline used in the study is openly accessible on GitHub at: [https://github.com/EttoreRocchi/MALDI-Kleb-AI].
