# Supplementary material for: Combining mass spectrometry and machine learning models for predicting Klebsiella pneumoniae antimicrobial resistance: a multicenter experience from clinical isolates in Italy
Source: BMC Microbiol. 2026 Jan 19;26:180. doi: 10.1186/s12866-025-04657-2 (PMC12952048; doi:10.1186/s12866-025-04657-2)
Supplement: Supplementary file 1 — Supplementary Material 1. [file 12866_2025_4657_MOESM1_ESM.pdf]

Supplementary Material for:

Combining Mass Spectrometry and Machine Learning Models for Predicting *Klebsiella pneumoniae* Antimicrobial Resistance: A Multicenter Experience from Clinical Isolates in Italy

Contents

S1 Batch effect correction using `combatlearn` **1**

S2 Detailed comparative analysis of machine learning models with aggregated datasets **3**

S3 Cross-site performance for amikacin **5**

S1 Batch effect correction using `combatlearn`

Batch effect correction was performed using the Python package `combatlearn` (v0.2.2), applying the `johnson` method for harmonization. This approach models batch-associated variability while preserving biologically meaningful signal and is specifically designed to avoid data leakage during harmonization, thereby maintaining the validity of downstream predictive modeling.

Visual inspection of UMAP embeddings (Figure S1) shows a clear reduction in site-specific separation after harmonization, with samples from different acquisition sites exhibiting increased overlap. To quantitatively assess the effectiveness of batch correction, we computed multiple complementary metrics, as detailed below.

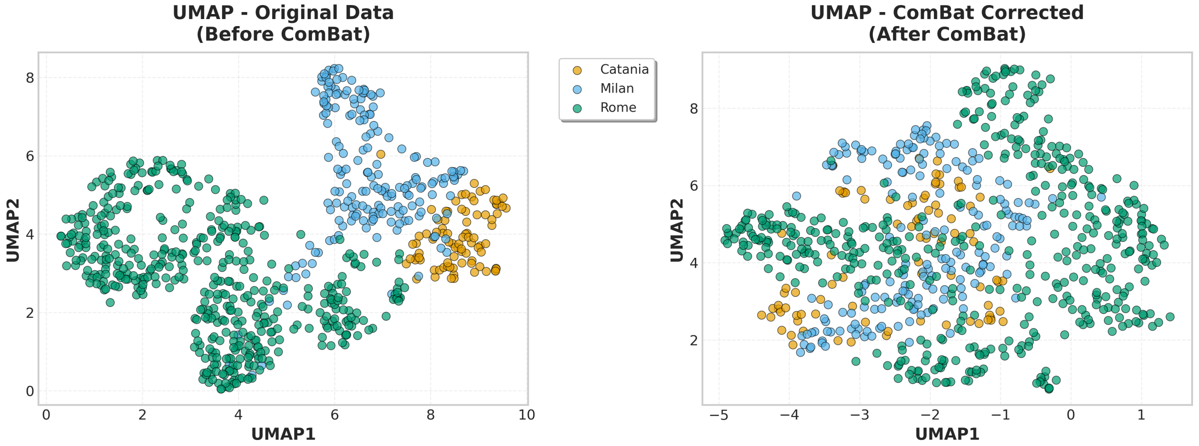

Figure S1: UMAP visualization of datasets before (left) and after (right) ComBat batch effect correction using `combatlearn`.

To evaluate batch mixing in low-dimensional representations, silhouette scores were computed using batch labels in both PCA and UMAP spaces. Before correction, positive silhouette scores (0.1725 in PCA space and 0.3340 in UMAP space) indicated moderate to high batch separability. After correction, silhouette scores became negative and near 0 (-0.0724 in PCA space and -0.0715 in UMAP space), consistent with effective batch mixing and the absence of systematic batch-driven clustering.

The preservation of intrinsic data structure after harmonization was evaluated at both local and global scales. Local structure was assessed using k-nearest neighbor (k-NN) overlap between the original and corrected feature spaces. Across neighborhood sizes (see Table S1), mean overlap values ranged from 0.66 to 0.60, with decreasing variance as k increased, indicating robust preservation of local relationships despite batch correction.

k	Mean Overlap	Std. Dev.
5	0.6600	0.2586
10	0.6444	0.2349
20	0.6142	0.2145
30	0.5976	0.1982

Table S1: k-NN neighborhood preservation between original and harmonized feature spaces.

Local structure preservation within each batch, assessed via k-NN overlap ($k = 10$), was high across all sites, with mean overlaps of 0.73 (Catania), 0.82 (Milan), and 0.86 (Rome), indicating that within-batch relationships were well preserved.

Global structure preservation was assessed using Spearman correlation between pairwise

distance matrices before and after harmonization. A high correlation coefficient ($\rho = 0.74$) was observed, demonstrating that the overall geometry of the data was largely maintained.

To quantitatively assess the impact of harmonization on feature distributions, changes in skewness and Kolmogorov–Smirnov (KS) statistics were computed. The median skewness change (0.1682) was modest and consistent with expectations for features with very small magnitudes (of the order of $\sim 10^{-4}$ or lower), for which minor location or scale adjustments can lead to measurable changes in distributional asymmetry. Similarly, KS statistics indicated moderate distributional changes (median: 0.2746), consistent with effective batch harmonization while avoiding excessive distortion of the original feature distributions.

S2 Detailed comparative analysis of machine learning models with aggregated datasets

Hyperparameter grids

To optimize each model, we explored the hyperparameter grids summarized in Table S2.

**Model**	**Hyperparameters**
Random Forest	`n_estimators`: [100, 200]<br>`criterion`: [gini, entropy]<br>`max_features`: [auto, sqrt, log2]<br>`max_depth`: [3, 5, 7]
Logistic Regression	`C`: [0.1, 1, 10, 100]<br>`penalty`: [l1, l2]
XGBoost	`booster`: [gbtree, dart]<br>`n_estimators`: [100, 200]<br>`max_depth`: [3, 5, 7]
MLP	`hidden_layer_sizes`: [(256,128), (128,64)]<br>`alpha`: [10^{-4}, 10^{-5}]

Table S2: Hyperparameter grids used for model optimization. Abbreviations: MLP = Multi Layer Perceptron

Comparative performance of macine learning models

The comparative performance of the four classifiers on the amikacin dataset is reported in Table S3.

Metric	Logistic Regression	MLP	Random Forest	XGBoost
Precision (0)	0.680 [0.633, 0.722]	0.696 [0.656, 0.737]	**0.742 [0.717, 0.767]**	**0.742 [0.702, 0.783]**
Precision (1)	0.708 [0.667, 0.745]	0.685 [0.644, 0.725]	0.712 [0.687, 0.738]	**0.722 [0.682, 0.765]**
Recall (0)	**0.739 [0.702, 0.776]**	0.676 [0.589, 0.743]	0.694 [0.659, 0.729]	0.710 [0.661, 0.763]
Recall (1)	0.642 [0.570, 0.704]	0.692 [0.612, 0.766]	**0.757 [0.731, 0.783]**	0.749 [0.706, 0.794]
F1 (0)	0.706 [0.671, 0.739]	0.677 [0.623, 0.721]	0.716 [0.691, 0.739]	**0.723 [0.686, 0.761]**
F1 (1)	0.670 [0.615, 0.716]	0.681 [0.636, 0.724]	0.732 [0.713, 0.751]	**0.733 [0.699, 0.768]**
MCC	0.384 [0.301, 0.457]	0.374 [0.307, 0.444]	0.452 [0.409, 0.492]	**0.461 [0.390, 0.533]**
Balanced Acc	0.691 [0.648, 0.727]	0.684 [0.649, 0.720]	0.725 [0.704, 0.745]	**0.729 [0.694, 0.765]**
AUROC	0.741 [0.705, 0.779]	0.739 [0.707, 0.774]	0.802 [0.779, 0.825]	**0.822 [0.799, 0.845]**

Table S3: Performance comparison of classification models on the amikacin dataset. Values are reported as mean performance with 95% confidence intervals estimated via bootstrap resampling (10000 iterations). Best-performing models for each metric are highlighted in bold. Class (0) corresponds to susceptible isolates, while class (1) corresponds to resistant isolates. Abbreviations: MLP = Multi-Layer Perceptron; MCC = Matthews Correlation Coefficient.

Similarly, Table S4 summarizes the predictive performance of the models on the meropenem dataset.

Metric	Logistic Regression	MLP	Random Forest	XGBoost
Precision (0)	**0.849 [0.832, 0.864]**	0.786 [0.770, 0.803]	0.769 [0.750, 0.787]	0.832 [0.823, 0.840]
Precision (1)	0.656 [0.600, 0.715]	0.724 [0.646, 0.799]	**0.946 [0.902, 0.986]**	0.855 [0.807, 0.899]
Recall (0)	0.823 [0.780, 0.867]	0.911 [0.875, 0.942]	**0.990 [0.982, 0.998]**	0.951 [0.929, 0.968]
Recall (1)	**0.684 [0.650, 0.719]**	0.465 [0.422, 0.510]	0.355 [0.293, 0.418]	0.586 [0.560, 0.611]
F1 (0)	0.835 [0.808, 0.860]	0.843 [0.821, 0.864]	0.865 [0.853, 0.878]	**0.887 [0.877, 0.896]**
F1 (1)	0.665 [0.631, 0.699]	0.562 [0.516, 0.612]	0.508 [0.440, 0.577]	**0.692 [0.673, 0.713]**
MCC	0.505 [0.447, 0.562]	0.437 [0.367, 0.509]	0.493 [0.436, 0.551]	**0.606 [0.574, 0.637]**
Balanced Acc	0.754 [0.727, 0.779]	0.688 [0.660, 0.719]	0.673 [0.642, 0.705]	**0.768 [0.756, 0.781]**
AUROC	0.829 [0.806, 0.853]	0.768 [0.743, 0.793]	0.870 [0.850, 0.892]	**0.887 [0.870, 0.900]**

Table S4: Performance comparison of classification models on the meropenem dataset. Values are reported as mean performance with 95% confidence intervals estimated via bootstrap resampling (10000 iterations). Best-performing models for each metric are highlighted in bold. Class (0) corresponds to susceptible isolates, while class (1) corresponds to resistant isolates. Abbreviations: MLP = Multi-Layer Perceptron; MCC = Matthews Correlation Coefficient.

S3 Cross-site performance for amikacin

The cross-site AUROC average values for amikacin prediction through XGBoost are reported in Figure S2.

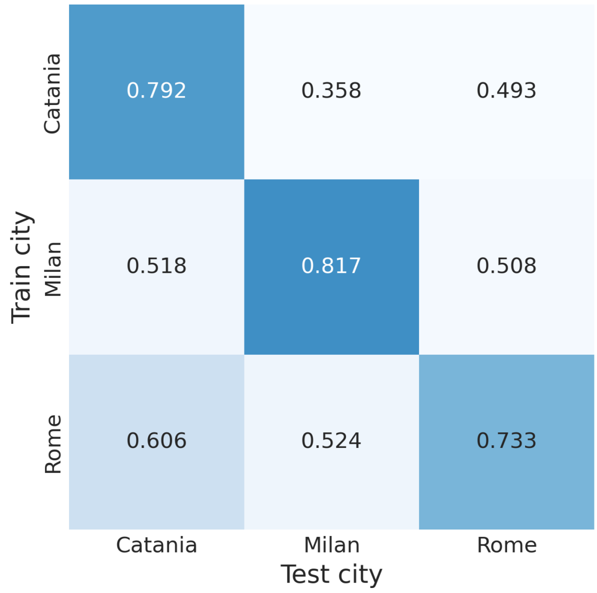

Figure S2: Cross-site AUROC heatmap for XGBoost trained and tested on different cities.
